# The 3D genome landscape: Diverse chromosomal interactions and their functional implications

**DOI:** 10.3389/fcell.2022.968145

**Published:** 2022-08-11

**Authors:** Katherine Fleck, Romir Raj, Jelena Erceg

**Affiliations:** ^1^ Department of Molecular and Cell Biology, University of Connecticut, Storrs, CT, United States; ^2^ Institute for Systems Genomics, University of Connecticut, Storrs, CT, United States; ^3^ Department of Genetics and Genome Sciences, University of Connecticut Health Center, Farmington, CT, United States

**Keywords:** 3D genome organization, gene regulation, intra-chromosomal contacts, inter-chromosomal contacts, meiotic chromosomes, homolog pairing, sister chromatids, development

## Abstract

Genome organization includes contacts both within a single chromosome and between distinct chromosomes. Thus, regulatory organization in the nucleus may include interplay of these two types of chromosomal interactions with genome activity. Emerging advances in omics and single-cell imaging technologies have allowed new insights into chromosomal contacts, including those of homologs and sister chromatids, and their significance to genome function. In this review, we highlight recent studies in this field and discuss their impact on understanding the principles of chromosome organization and associated functional implications in diverse cellular processes. Specifically, we describe the contributions of intra-chromosomal, inter-homolog, and inter-sister chromatid contacts to genome organization and gene expression.

## Introduction

The genome in eukaryotes is packaged and regulated intricately within the nucleus throughout development. The path from zygote to fully developed multicellular organism includes extensive genome remodeling to achieve diverse cell types. Thus, development represents a powerful system to investigate the processes that lead to varying cellular identities. These fascinating processes include changes at the level of 3D genome structure, epigenetic landscape, and transcription. However, the impact of these changes and their potential interplay remain a topic of active debate. Emerging technologies for high-throughput imaging and mapping of chromosomal contacts have enabled new insights into the relationship between genome morphology, function, and development. Such technological advances have been highlighted in previous reviews (Davies et al., 2017; Kempfer and Pombo, 2020; Hickey et al., 2021; Jerkovic and Cavalli, 2021; Rao et al., 2021). Here, we will discuss recent studies on how interactions between different chromosomes (inter-chromosomal) and those within individual chromosomes (intra-chromosomal) may bear functional significance in the regulation of various cell fates during development.

### The interplay between chromosome structure and function

Genome structure is highly organized at several levels of complexity. For instance, the formation of chromatin loops through extrusion can demarcate domains (also called topologically associating domains; TADs), and thereby, partition chromosomes (Rao et al., 2014; Sanborn et al., 2015; Fudenberg et al., 2016). Such domains represent regions of high contact frequency within insulated chromatin neighborhoods separated by boundary regions of low contact frequency (Dixon et al., 2012; Nora et al., 2012; Sexton et al., 2012; Rao et al., 2014). Furthermore, nuclear organization can be spatially segregated into two compartment types associated with either open or closed chromatin (Lieberman-Aiden et al., 2009). More globally, individual chromosomes can occupy distinct territories with a propensity to intermingle with neighboring chromosomes (Cremer and Cremer, 2001; Bolzer et al., 2005; Branco and Pombo, 2006).

Despite an emerging detailed genome structure, it is still unclear whether genome structure is a mere reflection of genome function or if regulation of gene expression is driven by genome organization, ultimately leading to cellular identities [recently reviewed in (van Steensel and Furlong, 2019; Ghavi-Helm, 2020; McCord et al., 2020; Oudelaar and Higgs, 2021)]. Specifically, the impact of chromosomal disruptions on chromatin organization and function seems to vary. For instance, structural disruptions at some individual loci result in a dramatic impact on gene regulation and disease (Spielmann et al., 2018; Ghavi-Helm, 2020; Oudelaar and Higgs, 2021). In contrast, other global and local chromosomal rearrangements do not appear to lead to major alterations in gene expression (Spielmann et al., 2018; Akdemir et al., 2020; Ghavi-Helm, 2020; Oudelaar and Higgs, 2021). Moreover, depletion of regulators such as cohesin and CTCF, both implicated in genome architecture, does not have a strong impact on gene expression (Nora et al., 2017; Rao et al., 2017; Schwarzer et al., 2017). These opposing findings have implications for our understanding of how certain factors act on distinct regulatory elements such as enhancers and promoters to orchestrate cell type-specific gene expression. Some mechanisms suggest chromosome looping may mediate enhancer-promoter contacts and could be correlated with gene activity (Palstra et al., 2003; Vernimmen et al., 2007; Rao et al., 2014; Bonev et al., 2017; Freire-Pritchett et al., 2017; Ghavi-Helm, 2020; Oudelaar et al., 2020; Oudelaar and Higgs, 2021; Reed et al., 2022). Interestingly, a recent study suggests that distinct regulatory sequences, termed tethering elements, could mediate distal enhancer-promoter contacts and determine activation dynamics (Batut et al., 2022). Such promoter-proximal tethering elements are also implicated in co-regulation of distant genes that have mutually shared enhancers (Levo et al., 2022). Conversely, direct contacts between enhancers and promoters may not be required to facilitate gene expression (Alexander et al., 2019; Benabdallah et al., 2019; Heist et al., 2019). Moreover, chromatin contacts do not seem to alter between different embryonic cell types regardless of changes in gene expression (Espinola et al., 2021; Ing-Simmons et al., 2021). Chromosome looping could also be involved in the formation of insulated chromatin domains within individual chromosomes (Dowen et al., 2014; Rao et al., 2014; Sanborn et al., 2015; Fudenberg et al., 2016; Hnisz et al., 2016; Ji et al., 2016). These insulated domains may facilitate enhancer-promoter contacts within domains and could prevent improper contacts between nearby neighboring domains (Dowen et al., 2014; Hnisz et al., 2016; Ji et al., 2016; Spielmann et al., 2018; Batut et al., 2022; Zuin et al., 2022). As promoters and enhancers drive gene expression in development, the generation of such insulated chromatin domains may be key for proper regulatory interactions.

### Functional implications of inter-chromosomal contacts

In addition to the spatial organization of individual chromosomes, positioning and interactions between different chromosomes have been gaining increasing attention due to their potential role in multiple cellular processes such as translocations, gene regulation, DNA repair, and evolution. For instance, in various systems, the levels of inter-chromosomal interactions relate to the frequencies of chromosomal translocations (Bickmore and Teague, 2002; Hlatky et al., 2002; Holley et al., 2002; Roix et al., 2003; Arsuaga et al., 2004; Branco and Pombo, 2006; Klein et al., 2011; Engreitz et al., 2012; Evdokimova et al., 2012; Zhang et al., 2012; Roukos et al., 2013; Canela et al., 2017; Rosin et al., 2019). These interactions are regulated by condensin II complex during interphase (Rosin et al., 2018). Consequently, condensin II knockdown results in increased translocation events in the presence of DNA damage (Rosin et al., 2019). Thus, proper intermingling of chromosome territories may be important in securing genome integrity from aberrant translocations. Appearance of elevated translocation events may have significant implications for diseases (Bickmore and Teague, 2002; Roix et al., 2003; Branco and Pombo, 2006; Klein et al., 2011; Engreitz et al., 2012; Evdokimova et al., 2012; Zhang et al., 2012; Roukos et al., 2013; Canela et al., 2017). Moreover, an increase of inter-chromosomal contacts among smaller chromosomes compared to larger ones across multiple vertebrate species may have indications for recombination rates and chromosome evolution (Tanabe et al., 2002; Lieberman-Aiden et al., 2009; Perry et al., 2021; Marlétaz et al., 2022).

Interactions between different chromosomes have also been implicated in gene regulation and chromatin segregation. Active regions associated with open chromatin and gene expression may be in close spatial proximity even if located on different chromosomes (Osborne et al., 2004; Branco and Pombo, 2006; Spilianakis and Flavell, 2006; Zhao et al., 2006; Apostolou and Thanos, 2008; Schoenfelder et al., 2010; Markenscoff-Papadimitriou et al., 2014; Maass et al., 2019; Monahan et al., 2019). For example, monoallelic olfactory receptor expression involves inter-chromosomal interactions between the chosen allele and a collection of intergenic enhancers bound by transcription factors (Markenscoff-Papadimitriou et al., 2014; Monahan et al., 2019). Such specific multi-chromosomal interactions during differentiation could drive the diversity of cellular identities. On the other hand, regions associated with repressed chromatin can also interact. For instance, inter-chromosomal interactions have been implicated in constitutive and facultative heterochromatin formation including telomere and centromere clustering (Dernburg et al., 1996; Mayer et al., 2000; Dimitri, 2004; Bantignies et al., 2011; Clowney et al., 2012; Sexton et al., 2012; Li et al., 2015; Stadler et al., 2017; AlHaj Abed et al., 2019; Erceg et al., 2019).

**FIGURE 1:**
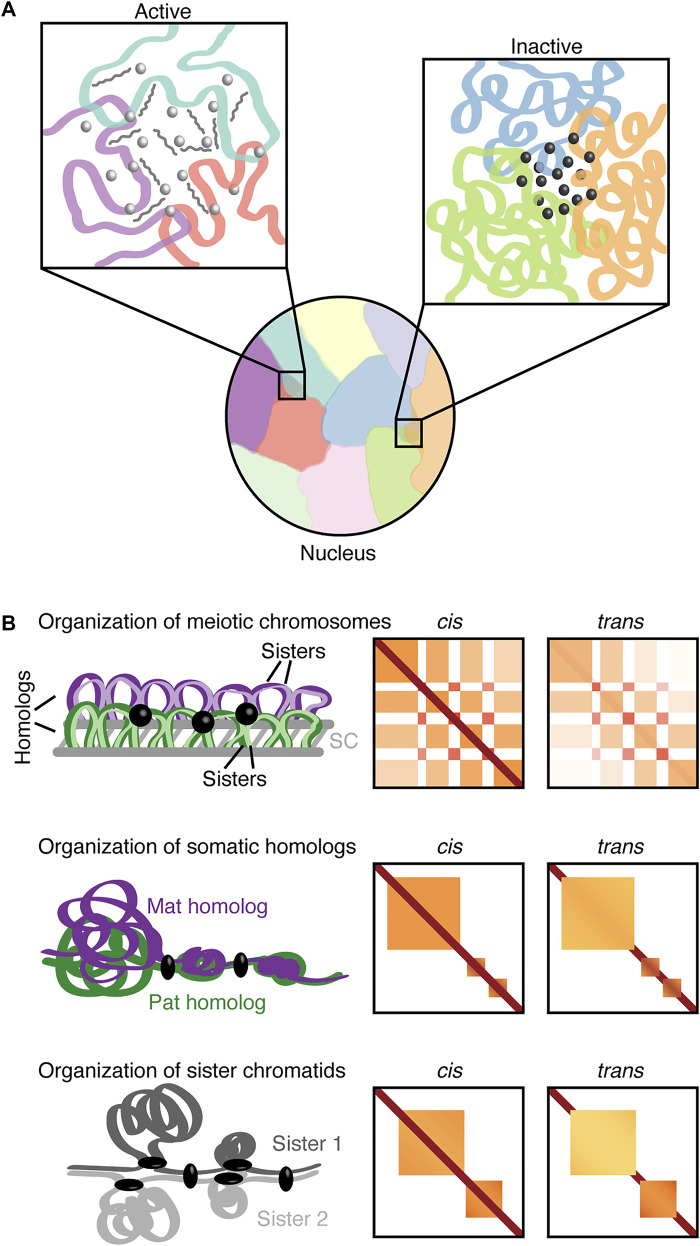
Nuclear inter-chromosomal interactions. **(A)** Chromosomes can occupy discrete territories with a tendency for neighboring chromosomes to intermingle. The left inset depicts an inter-chromosomal hub associated with open chromatin and active transcription with transcription factors (light gray spheres) and RNAs (medium gray). The right inset depicts an inter-chromosomal hub of heterochromatin with associated regulatory factors (dark gray spheres). **(B)** Schematic organization of mammalian meiotic chromosomes (pachynema; top panel), *Drosophila* somatic homologs (middle panel), and sister chromatids (bottom panel) with corresponding representations of *cis* and *trans* Hi-C contact maps. The meiotic *trans* contact map depicts inter-homolog contacts. Purple, maternal homolog (Mat); green, paternal homolog (Pat); respective shades of the homolog colors (top panel) or two shades of gray (bottom panel), sister chromatids; gray lines, synaptonemal complex (SC); black sphere, transcription-related clustering; black ellipsoid, regulators (insulators or architectural proteins).

The concentration of RNAs and proteins with their functionally related genomic loci in the nucleus has gained increasing attention (Bouwman et al., 2022). Specifically, phase separation and multivalent interactions have been implicated in the formation of active and inactive hubs [reviewed in Sabari et al. (2020)]. Complementarily, recent technological advances including ligation-independent approaches have provided insight into multi-way and inter-chromosomal interactions as well as integration of transcript and protein information (Kempfer and Pombo, 2020; Jerkovic and Cavalli, 2021; Winick-Ng et al., 2021). For instance, split-pool recognition of interactions by tag extension (SPRITE) and its derivatives extensively map inter-chromosomal hubs associated with both gene activation and silencing around distinct nuclear bodies (Quinodoz et al., 2018; Quinodoz et al., 2021; Arrastia et al., 2022). In the case of transcription, inhibition of nascent RNAs may affect RNA processing hubs (Quinodoz et al., 2021). Similarly, knockdown of satellite RNAs could impact pericentromeric regions, namely the assembly of a heterochromatic chromocenter (Quinodoz et al., 2021) as previously observed during early development (Casanova et al., 2013). Thus, inter-chromosomal hubs may be another possibility of how gene expression is mediated through a high concentration of non-coding RNAs and/or transcription factors (Figure 1A). Such functional inter-chromosomal hubs could facilitate sharing of spatially clustered resources to selectively promote specific cellular processes.

### 3D chromosome organization in meiotic events

A specific type of interchromosomal interactions in diploid organisms may involve homologous maternal and paternal chromosomes with nearly identical sequences (Figure 1B). Such interactions between pairs of homologs are termed homolog pairing. In meiosis, the juxtaposition between paired homologous chromosomes is facilitated by a proteinaceous structure called the synaptonemal complex. This event together with the formation of DNA double-strand breaks is implicated in proper interhomolog contacts and the promotion of meiotic recombination [reviewed in Zickler and Kleckner (1999), Handel and Schimenti (2010), Keeney et al. (2014)]. Meiotic recombination followed by chromosome segregation mediates the generation of diverse, functional haploid gametes during gametogenesis. Defects in meiotic events may lead to mis-segregation and aneuploidy, thus, impacting fertility and developmental disorders (Hassold et al., 2007; Handel and Schimenti, 2010).

Recent technological advances in contact mapping approaches have allowed for in-depth investigations of the meiotic chromosome organization (Muller et al., 2018; Alavattam et al., 2019; Patel et al., 2019; Schalbetter et al., 2019; Wang et al., 2019). These approaches reveal that during mammalian meiotic prophase domains tend to dissolve, while compartments related to transcription are readily visible (Alavattam et al., 2019; Patel et al., 2019; Wang et al., 2019). Specifically, the gene-rich, transcriptionally active A compartments may form strong inter-chromosomal contacts (Alavattam et al., 2019). Furthermore, haplotype-specific Hi-C has revealed that compartments and clustered transcription-related interactions also occur between paired homologs (Patel et al., 2019). Conversely, meiotic chromosomes condense into arrays of chromatin loops to facilitate effective chromosome segregation (Muller et al., 2018; Alavattam et al., 2019; Patel et al., 2019; Schalbetter et al., 2019; Wang et al., 2019). Such observations indicate that the balance between the compaction of meiotic chromosomes, homolog pairing, and transcription may be critical for development.

While compartments are associated with homolog pairing, the compartment type is also related to meiotic recombination (Patel et al., 2019). In particular, meiotic DNA double-strand break hotspots correlate with the gene-rich A compartments (Patel et al., 2019). In contrast to autosomal recombination, the sex chromosomes X and Y can only pair, synapse, and recombine in the small pseudoautosomal region. The remaining unsynapsed parts of X and Y chromosomes are subject to meiotic sex chromosome inactivation (MSCI) (McKee and Handel, 1993; Handel, 2004; Turner, 2007). Chromosomal contact maps reveal reorganization of the X chromosome from zygonema into pachynema, including depletion of compartments and transcription-related clustering (Alavattam et al., 2019; Patel et al., 2019; Wang et al., 2019). However, the meiotic compaction of X chromosome through chromatin loop arrays is retained (Patel et al., 2019; Wang et al., 2019). Notably, this X chromosome organization in male meiosis is distinct from the inactive X chromosome organization in female X-chromosome inactivation (XCI) (Nora et al., 2012; Rao et al., 2014; Deng et al., 2015; Minajigi et al., 2015; Darrow et al., 2016; Giorgetti et al., 2016; Bonora et al., 2018). This difference may be potentially related to different underlying mechanisms, such as DNA damage response in MSCI or non-coding transcript Xist in XCI (Ichijima et al., 2012; Loda et al., 2022).

The detailed structure of meiotic chromosomes may vary between species as checkerboard patterns on heatmaps have not yet been observed in yeast (Muller et al., 2018; Schalbetter et al., 2019). However, the principal chromatin organization of loop arrays emanating from a proteinaceous axis is preserved across species (Muller et al., 2018; Alavattam et al., 2019; Patel et al., 2019; Schalbetter et al., 2019; Wang et al., 2019). The observation that defects in the synaptonemal complex may impact chromosome compaction in different systems (Schalbetter et al., 2019; Wang et al., 2019) further supports the notion that the fundamental global organization of meiotic chromosomes is largely conserved.

### Varying structures and related roles of somatic homolog pairing

While the role of meiotic homolog pairing and its association with recombination is well studied (Zickler and Kleckner, 1999; Handel and Schimenti, 2010; Keeney et al., 2014; Patel et al., 2019), the precise role of somatic homolog pairing is still elusive. Homolog proximity was first noted more than a century ago (Stevens, 1908) and the potential influence between maternal and paternal homologs was hypothesized. Several decades later this communication was observed through interallelic complementation at the Bithorax complex (Lewis, 1954). Since then, this phenomenon, termed transvection, which involves pairing-dependent interallelic complementation, has been observed at multiple individual loci (Pirrotta, 1999; Wu and Morris, 1999; Duncan, 2002; Kennison and Southworth, 2002; McKee, 2004; Apte and Meller, 2012; Kassis, 2012; Blick et al., 2016; Joyce et al., 2016; Fukaya and Levine, 2017; Lim et al., 2018; Tian et al., 2019; Galouzis and Prud’homme, 2021). Homolog pairing can drive or silence gene expression through various regulatory elements including Polycomb response elements (PREs), insulators, enhancers, and promoters (Kassis et al., 1991; Fauvarque and Dura, 1993; Kassis, 1994; Gindhart and Kaufman, 1995; Kapoun and Kaufman, 1995; Geyer, 1997; Sigrist and Pirrotta, 1997; Fujioka et al., 1999; Muller et al., 1999; Zhou et al., 1999; Shimell et al., 2000; Duncan, 2002; Kennison and Southworth, 2002; Bantignies et al., 2003; Kravchenko et al., 2005; Vazquez et al., 2006; Fujioka et al., 2009; Li et al., 2011; Kassis, 2012; Blick et al., 2016; Fujioka et al., 2016; Joyce et al., 2016; Fukaya and Levine, 2017; Lim et al., 2018; Piwko et al., 2019; Galouzis and Prud’homme, 2021). Firstly, several specific factors were suggested to regulate pairing (Fritsch et al., 2006; Williams et al., 2007; Hartl et al., 2008), then comprehensive global screens were conducted to identify more factors implicated in somatic pairing (Bateman and Wu, 2008; Bateman et al., 2012b; Joyce et al., 2012). The identification of over one hundred factors that enhance or antagonize pairing indicates a delicate balance between pairing and anti-pairing of homologous chromosomes (Joyce et al., 2012; Joyce et al., 2016). These factors are implicated in key cellular processes such as mitotic cell division, DNA replication, and chromosome organization (Joyce et al., 2012). Interestingly, no zygotic product is required for pairing initiation in embryos (Bateman and Wu, 2008). Furthermore, over 90% of the identified factors are conserved from *Drosophila* to human (Joyce et al., 2012). In addition to extensive pairing in Dipteran insects such as *Drosophila*, pairing can also occur transiently at specific loci in mammals [reviewed in Apte and Meller (2012), Joyce et al. (2016)]. Such mammalian pairing has been observed in V(D)J recombination, DNA repair, imprinting, and XCI. In the latter case, pairing seems not to have a main impact on *Xist* regulation *in vitro*; whether pairing could influence XCI during development at other stages is still unclear (Barakat et al., 2014; Pollex and Heard, 2019; Loda et al., 2022).

Despite the implications of pairing in a plethora of cellular processes, the detailed structure of homolog pairing, and the global extent of its functional impact on gene regulation have been long-standing questions. Recent applications of advanced imaging technologies including sequential hybridization and super-resolution microscopy have revealed intricate structures of pairing. Specifically, *Drosophila* cell lines and embryos may include tightly paired regions and well-separated chromatin domains at a few homologous loci (Cattoni et al., 2017; Szabo et al., 2018; Cardozo Gizzi et al., 2019; Mateo et al., 2019). Alternatively, complementary strategies to microscopy, such as Hi-C-based approaches can reveal global and local pairing. For instance, Hi-C reads mapping to the same restriction fragments may facilitate the detection of short-range contacts between homologous chromosomes or sister chromatids (Rowley et al., 2019). Such an approach supports the enrichment of short-range chromosome pairing in active regions (Rowley et al., 2019). On the other hand, simulations that combined Hi-C with lamina-DamID suggest relationships between pairing strength and chromatin states (Li et al., 2017). Despite these predicted relationships, the challenge in distinguishing the homologous maternal and paternal chromosomes hampered the ability to elucidate pairing. Recent studies in *Drosophila* used haplotype-resolved Hi-C and developed a computational method, Ohm, to accurately distinguish *trans* contacts between homologous chromosomes from *cis* contacts within an individual homolog (AlHaj Abed et al., 2019; Erceg et al., 2019). Ohm also allowed for in-depth investigations of pairing ranging from kilobase to megabase scales. Together these studies (AlHaj Abed et al., 2019; Erceg et al., 2019) reveal that pairing is highly structured genome-wide with compartments, domains, and interaction peaks occurring between homologs. Pairing is also remarkably variable and composed of at least two modes; tightly paired regions with small domains alternating with domain boundaries and loosely paired regions with large single domains. Loose pairing is mainly associated with low gene expression and B compartments, while tight pairing may be associated with both lowly and highly transcribed genes, and largely A compartments (AlHaj Abed et al., 2019). Interestingly, most of the previously investigated transvection-related loci and the binding of insulator and architectural proteins (AlHaj Abed et al., 2019; Rowley et al., 2019) coincide with tightly paired regions (AlHaj Abed et al., 2019). Hence, varying structures of homolog pairing including tight and loose pairing in somatic cells can bear functional significance to gene expression. These observations provide unprecedented global connections of pairing structure with gene regulation.

### Homolog pairing during early development and differentiation

Since pairing is important for the regulation of gene expression, somatic pairing could be a key step in mediating the acquisition of cellular identities during development. For instance, pairing levels increase dramatically in development (Hiraoka et al., 1993; Fung et al., 1998; Gemkow et al., 1998; Joyce et al., 2013; Erceg et al., 2019). Specifically, this may indicate the role of somatic homolog pairing in the growth and development of organisms. Pairing levels are globally correlated with nascent gene expression and binding of RNA Pol II during zygotic genome activation (Erceg et al., 2019), a key event when the embryonic genome is activated. Depletion of the pioneer factor Zelda, which mediates chromatin accessibility in early embryogenesis, affects local levels of pairing (Erceg et al., 2019). Thus, establishment of homolog pairing is closely intertwined with genome activation and the opening of chromatin, where bringing homologs together may facilitate the formation of functionally compartmentalized inter-chromosomal hubs with concentrated regulatory elements and factors (Strom et al., 2017; Lim et al., 2018; Erceg et al., 2019). On the other hand, spatial segregation of hubs as well as the Rabl orientation of polarized centromeres and telomeres may reduce homolog search space and facilitate pairing (Hiraoka et al., 1993; Fung et al., 1998; Gemkow et al., 1998; Erceg et al., 2019; Child et al., 2021).

**FIGURE 2:**
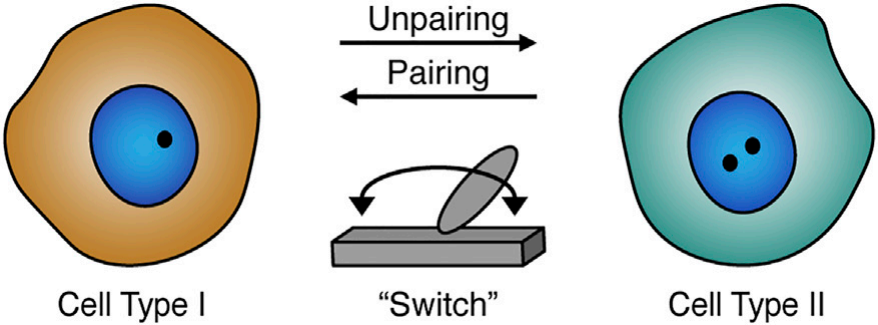
Homolog pairing role during development and differentiation. Homolog pairing levels may act as a potential “switch” (gray) that could play a role in cell type-specific gene regulation. The pairing status can also be related to cell differentiation including the formation or maintenance of cellular identities. Schematic of paired (one black dot) or unpaired (two black dots) homologs in the nucleus (blue).

In addition to somatic pairing in embryogenesis, pairing can also occur during *Drosophila* germline stem-cell differentiation preceding meiosis in the adult gonads (McKee, 2004; Christophorou et al., 2013; Joyce et al., 2013; Rubin et al., 2021; Antel et al., 2022). Interestingly, centromere pairing in differentiating mitotic cells prior to meiosis is dependent on the synaptonemal complex components, suggesting that pre-meiotic pairing may not be similar to somatic embryonic pairing (Christophorou et al., 2013; Joyce et al., 2013; Rubin et al., 2021). Differentiating cells also have decreasing levels of Stat92E expression, a factor that plays a role in maintaining stem cell identity (Sheng et al., 2009). The Stat92E locus has tight pairing interactions in germline stem cells (Antel et al., 2022). However, in differentiating gonialblasts the pairing immediately changes to loose, indicating that pairing may act as a “switch.” This “switch” may be dependent on cell specificity and could regulate transcription for a specific locus. Disturbances of the Stat92E pairing status can have an influential impact on its own gene expression, and consequently the differentiation of *Drosophila* germline (Antel et al., 2022). Another example suggests that changes in pairing levels of *Oct4* alleles in the mouse stem cell system are associated with a reduction in *Oct4* expression during the transition from pluripotent to differentiated state (Hogan et al., 2015). Together, these observations indicate a potential role of pairing in gene regulation during stem-cell differentiation (Hogan et al., 2015; Antel et al., 2022). In addition, pairing levels (Hiraoka et al., 1993; Dernburg et al., 1996; Fung et al., 1998; Gemkow et al., 1998; Joyce et al., 2013; Erceg et al., 2019) and transvection (Kassis et al., 1991; Bateman et al., 2012a; Mellert and Truman, 2012; Blick et al., 2016) can also vary in different cell types, including during development, where pairing levels may impact the effectiveness of the related transvection in the corresponding tissue (Viets et al., 2019). Thus, variation in levels of homolog pairing may facilitate cell type-specific gene regulation (Figure 2).

### Characteristics of sister chromatid organization

During DNA replication each homolog is replicated to generate a set of sister chromatids (Onn et al., 2008; Yatskevich et al., 2019), which adds another opportunity for interchromosomal interactions (Figure 1B). In *Drosophila* mechanisms behind homolog pairing, including those mediated by condensin II, could contribute to sister chromatid contacts (Senaratne et al., 2016). Interestingly, super-resolution imaging revealed that contacts between sister chromatids can manifest as distinct chromatin domains similar to homologs (Szabo et al., 2018). Moreover, live-cell and FISH imaging suggest that the separation of sister chromatids is associated with nuclear positioning, chromatin state, and replication timing (Stanyte et al., 2018). However, the global organization of sister chromatids remained largely elusive as sequence identity between sister chromatids presented a challenge for their distinction using typical sequencing-based methods. Recent studies have overcome this challenge by utilizing nucleotide analogs and then either chemical conversion to generate point mutations (Mitter et al., 2020) or Hoechst/ultraviolet treatment to degrade nucleotide-analog-incorporated strand (Oomen et al., 2020) followed by high-throughput sequencing. These approaches enabled detailed inspection of both *cis* interactions within individual sister chromatids and *trans* interactions between sister chromatids. Interestingly, in yeast, sister chromatids are precisely aligned at centromeres but display less aligned pairing along chromosome arms (Oomen et al., 2020). Meanwhile, in humans, the *trans* sister chromatid interactions are highly enhanced at domain boundaries (Mitter et al., 2020). In addition, the presence of *trans* interactions in domains varies depending on domain size. In smaller domains, which are associated with the Polycomb-repressive chromatin mark H3K27me3, sister chromatids are highly paired, whereas larger unpaired domains generally lack *trans* contacts and exhibit loose connections (Mitter et al., 2020). These *trans* sister chromatid interactions at domain boundaries and domains seem reminiscent of tightly and loosely paired regions observed in somatic homolog pairing (AlHaj Abed et al., 2019; Erceg et al., 2019). Nevertheless, distinct pools of cohesin complexes are implicated in global cohesion of aligned sister chromatids and local structuring of domains and boundaries during loop formation (Mitter et al., 2020; Oomen et al., 2020). Surprisingly, components of the cohesin complex are not identified in the screen for factors implicated in somatic homolog pairing (Joyce et al., 2012). These observations suggest that some of the underlying mechanisms for the pairing of sister chromatids and somatic homolog pairing may also differ.

Taken together, elucidating 3D organization of sister chromatids is a key step in understanding the role of sister chromatids in DNA repair, gene expression, and the cell cycle (Mitter et al., 2020; Oomen et al., 2020). More broadly, knowledge of principles of inter-chromosomal organization, including interactions between sister chromatids and those between homologs, will greatly enhance our fundamental understanding of their potential functional implications in diverse cellular processes.

## Concluding remarks

Broadly, variability can occur at any level of genome organization as suggested by single-cell omics and microscopy studies (Finn and Misteli, 2019). Single-cell approaches can highlight complex variability that could be missed by population-based approaches. Variability in the genome organization and gene expression of individual cells may provide plasticity in response to various stimuli (Finn and Misteli, 2019). To capture this plasticity, it is important to distinguish not only a handful of genes, but the dynamics of hundreds to thousands of genomic regions integrated with transcriptional activity. This facilitates investigations of individual cell fates within tissue microenvironments in response to developmental cues and disease progression. High-throughput imaging technologies have shown significant strides in bridging the gap from single cells to multicellular tissues using spatial genomics and transcriptomics (Kempfer and Pombo, 2020; Jerkovic and Cavalli, 2021; Rao et al., 2021; Zhao et al., 2022). Spatial-based DNA and RNA approaches integrated with burgeoning multiplexed protein imaging (Hickey et al., 2021; Takei et al., 2021; Ben-Chetrit et al., 2022; Liu et al., 2022; Vickovic et al., 2022) will enhance the understanding of how the dynamics of genome organization and function contribute to cellular identities.
